# The Value of Bead Coating in the Manufacturing of Amorphous Solid Dispersions: A Comparative Evaluation with Spray Drying

**DOI:** 10.3390/pharmaceutics14030613

**Published:** 2022-03-11

**Authors:** Eline Boel, Felien Reniers, Wim Dehaen, Guy Van den Mooter

**Affiliations:** 1 Department of Pharmaceutical and Pharmacological Sciences, Drug Delivery and Disposition, KU Leuven, 3000 Leuven, Belgium; eline.boel@kuleuven.be; 2 Department of Chemistry, Molecular Design and Synthesis, KU Leuven, 3001 Leuven, Belgium; felien.reniers@kuleuven.be (F.R.); wim.dehaen@kuleuven.be (W.D.)

**Keywords:** amorphous solid dispersion, bead coating, spray drying, drug loading screening, drug–polymer interactions, physical stability

## Abstract

Despite the fact that an amorphous solid dispersion (ASD)-coated pellet formulation offers potential advantages regarding the minimization of physical stability issues, there is still a lack of in-depth understanding of the bead coating process and its value in relation to spray drying. Therefore, bead coating and spray drying were both evaluated for their ability to manufacture high drug-loaded ASDs and for their ability to generate physically stable formulations. For this purpose, naproxen (NAP)–poly(vinyl-pyrrolidone-co-vinyl acetate) (PVP-VA) was selected as an interacting drug–polymer model system, whilst naproxen methyl ester (NAPME)–PVP-VA served as a non-interacting model system. The solvent employed in this study was methanol (MeOH). First, a crystallization tendency study revealed the rapid crystallization behavior of both model drugs. In the next step, ASDs were manufactured with bead coating as well as with spray drying and for each technique the highest possible drug load that still results in an amorphous system was defined via a drug loading screening approach. Bead coating showed greater ability to manufacture high drug-loaded ASDs as compared to spray drying, with a rather small difference for the interacting drug–polymer model system studied but with a remarkable difference for the non-interacting system. In addition, the importance of drug–polymer interactions in achieving high drug loadings is demonstrated. Finally, ASDs coated onto pellets were found to be more physically stable in comparison to the spray dried formulations, strengthening the value of bead coating for ASD manufacturing purposes.

## 1. Introduction

**Figure 1:**
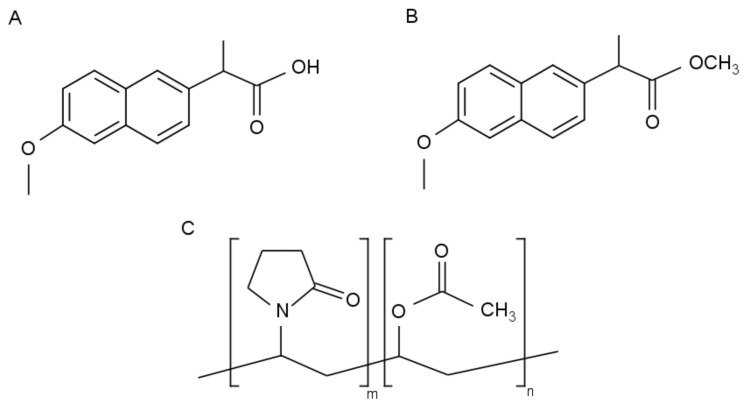
Structural formulas of (**A**) NAP, (**B**) NAPME and (**C**) PVP-VA.

At present, about 90% of new chemical entities (NCE) in drug discovery pipelines are characterized by a low aqueous solubility [1,2]. Aiming at oral administration, which can be considered the most popular and desired drug delivery route, many formulation strategies have been developed to enhance the oral bioavailability of these compounds [3]. One of the most successful approaches is to disperse a poorly water-soluble drug on a molecular level within an inert polymer matrix in the solid state, thereby creating an amorphous solid dispersion (ASD) [4,5]. In solid dispersions, the polymer acts as a stabilizer via anti-plasticizing effects, viscosity effects and potential drug–polymer intermolecular interactions to prevent drug–polymer phase separation and drug crystallization [6,7,8,9]. An ASD or glass solution is, however, only thermodynamically stable (i.e., the drug will never crystallize), in case the drug content is below its thermodynamic solubility limit in the polymer [10,11]. The persisting trend to lower the pill burden to promote patient therapeutic compliance requires the implementation of as high as possible drug loadings within the polymer [5,12,13]. Such high drug-loaded ASDs can imply physical stability issues during down-stream processing, as well as during storage, and can therefore defeat the ASD solubility advantage [2]. Furthermore, physical stability problems are often regarded as one of the main reasons why only a limited number of ASDs have been commercialized so far [4,5,14]. From this point of view, the one-step bead coating or fluid-bed coating process offers interesting prospects to manufacture ASDs in contrast to spray drying by avoiding subsequent pre-densification or compression steps that might promote crystallization [6,15]. Although considerable research has been devoted to spray drying for ASD manufacturing purposes, rather less attention has been paid to bead coating. Moreover, only a few studies have compared bead coating with other solid dispersion preparation techniques, which implies that its potential value within the ASD formulation platform is still unclear. For example, Kim et al. made a comparative assessment between bead coating and spray drying for the preparation of controlled-release microparticles in orally disintegrating tablets, mainly directing in vitro drug dissolution [16]. Concerning ASDs, bead coating has been compared with hot melt extrusion by Verreck et al.; however, the authors merely focused on drug release and bioavailability [17]. In the study of Lugtu-Pe et al., the effect of dosage form design on drug release profiles was investigated for the development of controlled release ASDs. Membrane-coated beads showed greater potential for sustained release than spray dried powders, implying a lower risk for solution-mediated phase transformation (i.e., crystallization during dissolution) [18]. From the abovementioned examples, it is clear that the focus is mainly on drug release, hence the current scientific literature does not contribute to an in-depth understanding of the physical chemistry behind the bead coating process and its resulting coated pellet formulations [15]. In one of our previous studies, bead coating and spray drying were evaluated for their ability to manufacture high-drug loaded miconazole (MIC)–poly(vinyl-pyrrolidone-co-vinyl acetate) (PVP-VA) ASDs [15]. However, a direct comparison between bead coating and spray drying to manufacture high drug-loaded ASDs comprising a fast crystallizing compound in combination with a physical stability evaluation of the resulting formulations has, to the best of our knowledge, never been carried out before. This study therefore aims to address these shortcomings, by first defining the highest possible drug load that still results in an amorphous system for both manufacturing techniques. For this purpose, naproxen (NAP)–PVP-VA was selected as an interacting drug–polymer model system, whilst naproxen methyl ester (NAPME)–PVP-VA served as a non-interacting drug–polymer model system (see Figure 1). For both solvent-based ASD manufacturing techniques studied, the solvent employed was methanol (MeOH). Additionally, the crystallization tendency of the model drugs was evaluated. In a next step, the interacting NAP–PVP-VA systems were further examined with respect to their physical stability for both preparation techniques.

## 2. Materials and Methods

### 2.1. Materials

NAP (i.e., 2-(6-Methoxynaphthalen-2-yl)propanoic acid) was obtained from SA Fagron NV (Waregem, Belgium). NAP was also used as substrate in the synthesis of NAPME (i.e., methyl 2-(6-Methoxynaphtalen-2-yl)propanoate (see Section 2.2). BASF ChemTrade GmbH (Ludwigshafen, Germany) supplied Kollidon-VA 64 (PVP-VA). Phosphorus pentoxide and MeOH (purity ≥ 99.8%) were purchased from Acros Organics (Geel, Belgium). Microcrystalline cellulose (MCC) pellets (Vivapur^®^ 700: 18–25 mesh, 710–1000 µm) were acquired from JRS Pharma GmbH (Rosenberg, Germany). ChemLab NV (Zedelgem, Belgium) provided sodium chloride.

### 2.2. Synthesis of Naproxen Methyl Ester

For the synthesis of naproxen methyl ester, 2-(6-Methoxynaphthalen-2-yl)propanoic acid, *p*-toluenesulfonic acid (ChemLab NV, Zedelgem, Belgium), 2,2-dimethoxypropane (Acros Organics, Geel, Belgium) and MeOH (purity ≥ 99.8%, Fisher Scientific, Loughborough, UK) were added to a round-bottom flask. The reaction mixture was refluxed for 18 h, followed by cooling to −20 °C. The formed crystals were filtered off and washed three times with cold MeOH. The pure product was obtained as a white solid in 91% yield. The detailed synthesis procedure, as well as nuclear magnetic resonance (NMR) and high-resolution mass spectrometry (HRMS) data, are presented in the Supporting Information. The spectra were in accordance with available literature data [19].

### 2.3. Crystallization Tendency Study

The crystallization tendency of both model compounds was evaluated in MeOH by means of a Büchi mini spray dryer B-191 (Büchi, Flawil, Switzerland). Accurate amounts of NAP were dissolved in 20.0 mL solvent, resulting in a solid content of 1.29% *w/v*. Its methyl ester was dissolved in 10.0 mL MeOH with a solid content of 1.37% *w*/*v*, thereby ensuring equal molarity. The solutions were prepared in triplicate and subsequently spray dried, with the drying air temperature set at 65 °C (corresponding to the boiling point of MeOH), the drying air flow rate at 33 m^3^/h, the atomization air flow rate at 15 L/min and a feed solution flow rate of 5 mL/min. The resulting spray dried samples were analyzed with modulated differential scanning calorimetry (mDSC) right after spray drying (day 0), after one day (day 1) and after one week (day 7) of storage in ambient conditions.

### 2.4. Manufacturing of Amorphous Solid Dispersions

#### 2.4.1. Spray Drying

Within the scope of the drug loading screening, accurate amounts of either NAP or NAPME, in combination with PVP-VA, were dissolved in 20.0 mL MeOH to obtain a solid content of 10% *w*/*v*. The same solid content but a 100.0 mL solvent amount was applied to prepare spray-dried NAP–PVP-VA for repeatability and physical stability assessment. The drug–polymer solutions were spray dried using a Büchi mini spray dryer B-191 (Büchi, Flawil, Switzerland) operating with the same process parameters as applied for the crystallization tendency study, apart from the atomization air flow rate, which was set at 10 L/min. The resulting spray dried ASDs were further dried in a vacuum oven (Mazzali Systems, Monza, Italy) at room temperature for 72 h. After this secondary drying step, the spray dried formulations were stored at −28 °C in the presence of phosphorus pentoxide until further analysis (within one day). The samples were analyzed as such with mDSC, X-ray powder diffraction (XRPD) and scanning electron microscopy (SEM) to characterize their phase behavior, and with thermogravimetric analysis (TGA) to determine residual solvent levels.

#### 2.4.2. Bead Coating

Drug–polymer solutions, comprising either NAP or NAPME in combination with PVP-VA, with a solid content of 10% *w*/*v* in 200.0 mL MeOH, were coated onto 150.0 g MCC beads with a Mini-Glatt fluid bed coater (Glatt, Binzen, Germany) in a bottom spray setup. The coater is equipped with a Würster insert, with the partition height set at 7.5 mm. In a first step, the MCC beads were preheated via fluidization at 25 m^3^/h for 45 min, with the inlet temperature (T_inlet_) set at 50 °C (i.e., 15 °C below the boiling point of MeOH). Afterwards, the drug–polymer solutions were coated onto the beads with the following parameters applied: a feed rate of 0.76 mL/min, an atomization air pressure of 1 bar, a drying air flow kept between 33 and 35 m^3^/h, a tapping frequency installed at 5 s and a T_inlet_ set at 50 °C. This T_inlet_ setting ensures a bed temperature (T_bed_) of approximately 40 °C during the coating procedure. The ASD-coated pellets were fluidized for an additional 5 min before unloading and subsequently further dried in a vacuum oven (Mazzali Systems, Monza, Italy) at room temperature for 72 h. Afterwards, the formulations were stored at −28 °C in the presence of phosphorus pentoxide until further analysis (within one day). Coated pellets were milled with a laboratory cutter mill (Ika, Staufen, Germany) for 10 s to obtain fine powder that allows optimal thermal contact during mDSC and TGA measurements. Potential milling-induced crystallinity was evaluated with XRPD and SEM by analyzing the milled samples as well as the coated pellets as such.

### 2.5. Physical Stability Study

A physical stability study was performed on spray dried NAP–PVP-VA formulations and on NAP–PVP-VA-coated pellets. More specifically, the formulations with the highest possible NAP load per manufacturing technique (i.e., 40 wt% for bead coating and both 35 wt% and 40 wt% for spray drying) were evaluated. Spray dried powders and coated pellets as a whole were stored in a desiccator at different conditions related to temperature and relative humidity (RH), namely, 4 °C/0% RH, 40 °C/0% RH and 40 °C/75% RH. The 0% RH conditions were guaranteed by the presence of phosphorus pentoxide, whilst 75% RH was installed via a saturated sodium chloride solution. The phase behavior of the formulations was monitored with mDSC and XRPD for a period of three months. TGA was applied to verify the amount of residual solvent in the formulations at time point zero. Again, spray dried formulations were analyzed as such, while coated pellets were milled after the respective storage time and potential milling-induced crystallinity was evaluated with XRPD and SEM.

### 2.6. Solid-State Characterization

#### 2.6.1. Modulated Differential Scanning Calorimetry (mDSC)

The phase behavior of the formulations was evaluated with a Discovery DSC 2500 (TA Instruments, Leatherhead, UK) equipped with a refrigerated cooling system (RCS 90) under a dry nitrogen purge at a flow rate of 50 mL/min. Indium and sapphire standards were used in the calibration for temperature, enthalpy and heat capacity, respectively. For the mDSC measurements, a linear heating rate of 2 °C/min was combined with a modulation amplitude of 0.212 °C and a period of 40 s. Approximately 1–3 mg of the samples were accurately weighed in standard aluminum DSC pans (TA Instruments, Zellik, Belgium) and subsequently crimped with standard aluminum DSC lids (TA Instruments, Zellik, Belgium). Within the scope of the crystallization tendency study, spray dried NAP and spray dried NAPME were heated from 0 °C to 180 °C and from −40 °C to 120 °C, respectively. ASD formulations containing NAP, as part of the drug loading screening as well as the repeatability and physical stability assessment, were isothermally held at 40 °C for 30 min, followed by a heating procedure ranging from −10 °C to 180 °C. ASD formulations involving NAPME were also isothermally held at 40 °C for 30 min but were followed by a heating procedure from −60 °C to 140 °C. DSC thermograms were analyzed using Trios software (Version 5.1, TA Instruments, Leatherhead, UK). Glass transition temperatures (T_g_) were measured at half height of transition in the reversing heat flow (RHF). Calculations of crystallinity percentages were executed by means of melting enthalpy values.

#### 2.6.2. X-ray Powder Diffraction (XRPD)

XRPD was performed using an X’Pert PRO diffractometer (PANalytical, Almelo, The Netherlands) with a Cu tube (λ_Kα1_ = 1.5418 Å) and a generator installed at 45 kV and 40 mA. Measurements were executed at room temperature in transmission mode, using Kapton^®^ Polyimide Thin-films (PANalytical, Almelo, The Netherlands). For the highest possible drug loading determinations, the following experimental settings were selected: a continuous scan mode from 4° to 40° 2θ with 0.0167° step size and 400 s counting time. Within the scope of the repeatability assessment and physical stability study, a continuous scan mode from 17° to 21° 2θ with 0.0167° step size and a counting time of 2500 s was applied. The diffractograms were analyzed using the X’Pert Data Viewer (Version 1.9a, PANalytical, Almelo, The Netherlands). Crystallinity percentage calculations (i.e., degree of crystallinity relative to pure NAP) pertaining to the physical stability assessment were performed based on the area under the curve (AUC) of the highest intensity Bragg peak (i.e., at 19° 2θ), normalized by weight, by means of Origin (Version 8.6 series number GF3S4-9089-7123456, OriginLab Corporation, Northampton, MA, USA).

#### 2.6.3. Scanning Electron Microscopy (SEM)

SEM was performed to detect possible crystals and to evaluate potential milling-induced crystallinity for coated pellets. Therefore, samples were adhered to SEM stubs using double-sided carbon tape (Ted Pella Inc., Redding, CA, USA) and were gold-coated under vacuum with a SCD-030 Balzers Union sputter-coater (Oerlikon Balzers, Balzers, Liechtenstein) for 45 s at 20 mA. A Philips XL30 SEM-FEG (Philips, Eindhoven, The Netherlands), equipped with a Schottky field emission electron gun (beam of 5 to 20 kV) and a conventional Everhart–Thornley secondary electron detector, was used to record the images.

#### 2.6.4. Thermogravimetric Analysis (TGA)

A thermogravimetric analyzer 550 (TA Instruments, Leatherhead, UK) was applied to record weight loss (due to solvent evaporation) as a function of time to determine residual solvent levels of the formulations. Approximately 4–10 mg of the samples were weighed in a platinum pan (TA Instruments, Zellik, Belgium) and subsequently heated at 5 °C/min to 130 °C in ambient atmosphere. Resulting TGA profiles were analyzed using Universal Analysis software (Version 5.5, TA Instruments, Leatherhead, UK).

#### 2.6.5. Attenuated Total Reflectance Fourier Transform Infrared Spectroscopy (ATR-FTIR)

To confirm the presence and absence of drug–polymer specific H-bond interactions in the NAP–PVP-VA and NAPME–PVP-VA model system, respectively, ATR-FTIR was performed. FTIR spectra were collected at room temperature by means of a Vertex 70 FTIR spectrometer (Bruker, Billerica, USA) equipped with a platinum ATR accessory. The spectra were recorded in the range between 400 cm^−1^ and 4000 cm^−1^ with 64 scans at a spectral resolution of 4 cm^−1^. A background spectrum was collected under the same conditions and subtracted from each subsequent sample spectrum. FTIR spectra are presented in the Supporting Information (see Figures S1 and S2).

## 3. Results and Discussion

### 3.1. Crystallization Tendency Study

**Figure 2:**
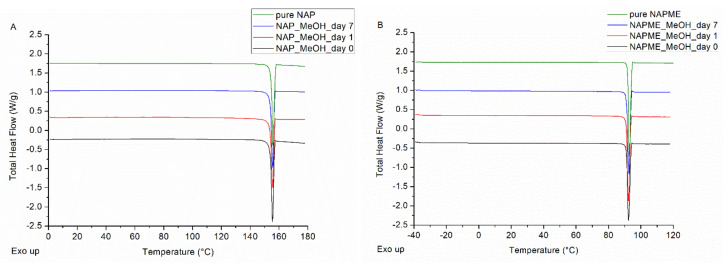
mDSC thermograms of (**A**) NAP spray dried from MeOH and (**B**) NAPME spray dried from MeOH, right after spray drying (black), after one day (red) and after one week (blue) of storage, and the mDSC thermogram of pure NAP and NAPME (green) as comparison. Total heat flow (THF) signals are shown as arbitrary units.

In a first step, the crystallization tendency of the two model drugs was examined, and both NAP and NAPME were found to exhibit fast crystallization. As shown in Figure 2, the mDSC thermograms of NAP and NAPME spray dried from a MeOH solution are only characterized by a melting event. Corresponding average crystallinity percentages, reported in Table 1, already amounted to 97.31% and 94.30% at day 0 for NAP and its methyl ester, respectively. Regarding the classification set forth by Van Eerdenbrugh et al., it can thus be concluded that both model compounds can be classified as glass forming ability (GFA) Class I compounds in MeOH [20,21]. In the literature, NAP is manifold defined as a fast crystallizer and, moreover, its high crystallization tendency appears to be irrespective of the solvent applied, as previously reported by our research group [11,22,23,24]. The rapid crystallization behavior observed for NAPME is, however, in contrast with the findings of Minecka et al. [22]. In this study, crystallization tendency impaired by substituting the hydrogen atom of the hydroxyl group of NAP. The fact that a significantly lower melting point (T_m_) was obtained for their synthesized methyl ester might serve as possible explanation. NMR and HRMS data, as well as a DSC purity analysis related to our synthesized NAPME, are presented in the Supporting Information.

### 3.2. Determination of the Highest Possible Drug Loading

The ability of bead coating and spray drying to manufacture high drug-loaded ASDs was assessed via a drug loading screening approach. For both techniques and both drug–polymer model systems, the highest possible drug loading that still results in an amorphous system was defined.

#### 3.2.1. NAP–PVP-VA Formulations

**Figure 3:**
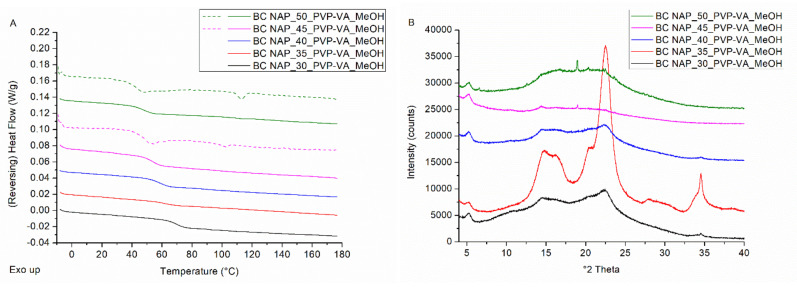
(**A**) mDSC thermograms of milled NAP–PVP-VA-coated beads (BC), with drug loadings from 30% (black) to 50% (green) by intermediate steps of 5%. The THF (dashed) and RHF (solid) signals are shown as arbitrary units. (**B**) Corresponding XRPD diffractograms. The intensities are shown as arbitrary units.

**Figure 4:**
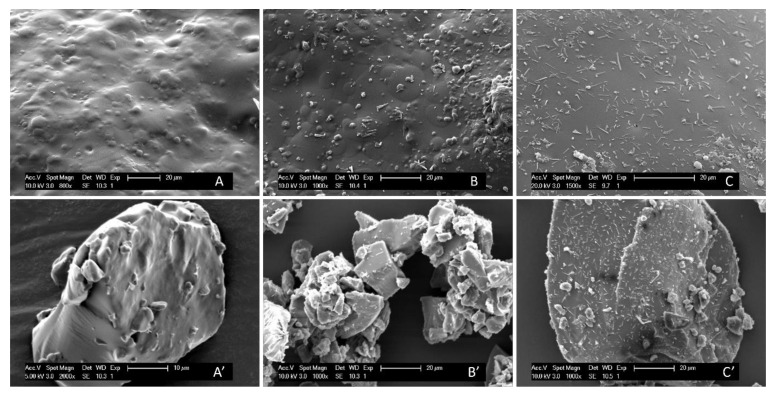
SEM images of the surface of NAP–PVP-VA-coated pellets with (**A**) 40% NAP load, (**B**) 45% NAP load and (**C**) 50% NAP load. SEM images of milled NAP–PVP-VA-coated pellets with (**A’**) 40% NAP load, (**B’**) 45% NAP load and (**C’**) 50% NAP load.

Initially, a drug loading screening was carried out on NAP–PVP-VA-coated pellets and the results are visualized in Figure 3. A NAP load (i.e., drug weight fraction) of 30% was applied as a starting point and consecutively increased by steps of 5 wt%. The mDSC thermograms (see Figure 3A) of milled NAP–PVP-VA-coated pellets with NAP weight fractions of 30–40 wt% show a single T_g_ event, indicating the formation of a one-phase amorphous system. From 45 wt% on, an endothermal melting event appears that points out the presence of crystalline NAP. For the formulation with 45% drug weight fraction, a T_m,average_ of 97.2 °C was found (associated with 1.4% crystalline content) and for the 50% drug loading formulation, a T_m,average_ of 108.7 °C was detected (associated with 3.0% crystalline content), as reported in Table 2. The increasing T_m,average_ with increasing drug load in the formulation is attributed to the lower amount of polymer able to dissolve NAP upon heating during the mDSC measurement, hence less melting point depression [25]. By comparison, the T_m_ of pure NAP was found to be 154.4 °C (see Figure 2A). Corresponding XRPD diffractograms (Figure 3B) reveal distinct Bragg peaks from 45% NAP loading on and are thus in line with the mDSC results. The other, much broader peaks that can be observed on the diffractograms, predominantly for the formulation with 35 wt% NAP, are attributed to the presence of MCC. In this case, milling resulted in an enlarged MCC core fraction as compared to the ASD coating fraction, and this also explains the less pronounced T_g_ event on the corresponding mDSC thermogram. XRPD analysis of the coated pellets as such resulted in an amorphous halo for all drug weight fractions studied (data not shown). The potential occurrence of milling-induced crystallinity was therefore further examined with SEM, by capturing images of coated beads as such, as well as of milled coated beads. As depicted in Figure 4, the images reveal the presence of needle-shaped NAP crystals from 45% drug loading on for both conditions. It is, however, difficult to interpret whether the NAP crystals in the milled samples are more pronounced as compared to the NAP crystals on the beads’ surface, hence it is inappropriate to form a conclusion about whether or not milling-induced crystallization occurred. Nevertheless, since NAP crystals could be visualized on the surface of NAP_45_PVP-VA-coated pellets (see Figure 4B), 40% can clearly be determined as the highest possible drug loading for NAP–PVP-VA systems manufactured with bead coating.

**Figure 5:**
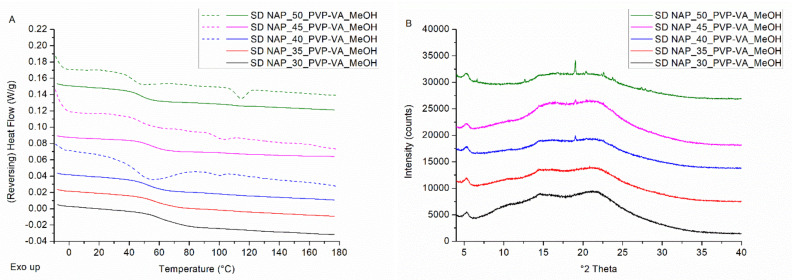
(**A**) mDSC thermograms of spray dried NAP–PVP-VA systems (SD), with drug loadings from 30% (black) to 50% (green) by intermediate steps of 5%. The THF (dashed) and RHF (solid) signals are shown as arbitrary units. (**B**) Corresponding XRPD diffractograms, where the intensities are also shown as arbitrary units.

In the same manner, a drug loading screening was performed for spray dried NAP–PVP-VA formulations (see Figure 5). A melting endotherm, corresponding to the presence of NAP crystals, can be identified on the mDSC thermograms from 40% NAP load on. To specify, for this drug weight fraction, a T_m,average_ of 93.5 °C was found that is associated with 1.1% crystallinity (see Table 2). Note, also, that for the spray dried samples, the T_m,average_ increases with increasing drug load. Corresponding XRPD diffractograms are characterized by distinct Bragg peaks from 40% drug weight fraction on, hence they are again in line with the mDSC data. Furthermore, SEM images correlate well with these outcomes, as needle-shaped NAP crystals could be visualized in the spray dried powders from 40% NAP load on (data not shown). Consequently, 35 wt% can be defined as the maximum achievable NAP loading for spray dried NAP–PVP-VA formulations.

It can thus be concluded that for the interacting drug–polymer model system studied, bead coating shows a greater ability to manufacture high drug-loaded ASDs; however, the observed difference between the two ASD preparation techniques is rather small. It is important to note that, for both manufacturing techniques, the highest drug loadings exceed the thermodynamic solid solubility of NAP in PVP-VA, which is reported to be 20.7 wt% [10,26]. This solubility limit was calculated by means of the thermodynamic perturbed-chain statistical associating fluid theory (PC-SAFT) model based on the solid–liquid equilibrium [26]. NAP weight fractions exceeding this thermodynamic solubility limit can be explained by kinetic stabilization effects, namely, impeding molecular mobility by immobilization of high NAP concentrations into a highly viscous polymer matrix [10,27]. During spray drying, the very fast solvent evaporation (i.e., in the millisecond range) kinetically traps the microstructure of the drug–polymer system, thereby preventing phase separation [28,29,30]. To clarify, the drug is susceptible to crystallization during solvent evaporation from the time it reaches its solubility limit in the solvent applied until immobilization into the highly viscous matrix. Slower solvent evaporation processes expand this timeframe, more readily allowing nuclei formation and successive crystal growth, especially at high drug loads [24,29,31]. One might thus expect a lower extent of kinetic stabilization for the bead coating process, as the atomized droplets reach the pellets with a sufficient solvent amount left to ensure spreading over the pellet surface and subsequent film formation [32]. Notwithstanding, skin formation due to very fast solvent evaporation can result in solvent entrapment, which in turn might promote crystallization [24,30]. Moreover, Paudel et al. found that spray drying process parameters leading to a higher rate of solvent evaporation resulted in amorphous–amorphous phase separated NAP–polyvinylpyrrolidone (PVP) K25 systems [31]. The authors describe that for slower solvent evaporation conditions, there is sufficient time for polymer conformational fluctuations, which favors the molecular mixing process [31]. In the research of Kojima et al., when preparing solid dispersions with spray drying under fast solvent evaporation conditions, agglomeration at the surface of the drying droplets occurred due to a lack of time to diffuse into the core [33]. This resulted in a heterogeneous, phase-separated distribution of drug and polymer and consequently in low solubility behavior and physical instability. On the other hand, spray drying under slower evaporation conditions ensured a homogeneous distribution of solute molecules [33]. These findings are in accordance with our observations, namely, that slightly higher NAP loads could be achieved with bead coating, even though spray drying (i.e., T_inlet_) operated at the boiling point of the solvent and the T_bed_ during the bead coating procedure was about 40 °C. Moreover, the rate of solid phase crystallization can also be related to the difference between the product temperature (i.e., T_bed_ for bead coating and outlet temperature (T_outlet_) for spray drying) and the T_g_ of the system [34]. In general, smaller differences were obtained for spray drying because of the higher value of T_outlet_ compared to that of the resulting product T_g_, which might contribute to an increased NAP crystallization tendency. To exemplify, the difference between the T_bed_ and the T_g_ of NAP_40_PVP-VA prepared with bead coating is approximately 17 °C, while the difference between the T_outlet_ and the T_g_ of NAP_40_PVP-VA prepared with spray drying amounts to only 9 °C. The fact that the difference in maximum achievable drug loading between the two ASD preparation techniques is rather small can be explained by the presence of drug–polymer interactions in combination with the short timeframe over which solvent evaporation occurs [25]. FTIR spectra were in accordance with available literature data and hence confirm that NAP and PVP-VA form an interacting system via hydrogen bonds (see Figure S1, Supporting Information) [35].

More detailed results related to the phase behavior of coated and spray dried NAP–PVP-VA formulations are reported in Table 2. It is evident that T_g_ values decrease with increasing NAP fractions. For the spray dried samples, T_g_ values are systematically lower than the ones obtained for the milled ASD-coated pellets, which can, however, not be linked to their according residual solvent percentages. This might, at least partially, be due to small differences in NAP content. Interestingly, T_g_s are generally broader for the spray dried samples, implying that these systems are more heterogeneous in comparison with the ASDs deposited onto pellets. For the spray dried formulations, T_g_ width seems to decrease with increasing NAP fraction, which may be due to the more narrow T_g_ of NAP relative to that of PVP-VA.

**Table 2:** Overview of the phase behavior of NAP–PVP-VA formulations prepared with bead coating and spray drying. Average T_g_s, T_g_ widths, T_m_ values and crystallinity percentages ± sd were obtained from mDSC measurements. Residual solvent percentages were derived from TGA analyses. NA = not applicable. The T_g_ of pure PVP-VA was determined to be 110.3 °C (second heating cycle), while the T_g_ of pure NAP is reported to be 5 °C [36].

Drug Load	Average T_g_ ± sd (°C)	Average T_g_ Width ± sd (°C)	Average T_m_ ± sd (°C)	Average Crystalline Content ± sd (%)	Residual Solvent (%)
Bead coating					
30%	69.0 ± 0.7	10.5 ± 1.7	NA	NA	2.0
35%	62.9 ± 1.0	11.7 ± 1.7	NA	NA	2.8
40%	58.4 ± 0.3	11.4 ± 0.6	NA	NA	1.0
45%	53.6 ± 0.4	11.2 ± 1.3	97.2 ± 0.3	1.4 ± 0.06	0.8
50%	49.8 ± 2.6	13.0 ± 6.4	108.7 ± 0.9	3.0 ± 1.1	1.0
Spray drying					
30%	63.5 ± 0.9	24.8 ± 0.7	NA	NA	1.8
35%	59.2 ± 1.0	20.8 ± 0.5	NA	NA	1.6
40%	55.2 ± 2.0	17.1 ± 0.4	93.5 ± 1.4	1.1 ± 0.2	1.3
45%	50.3 ± 4.6	17.1 ± 1.2	99.9 ± 2.9	2.1 ± 1.7	1.1
50%	48.2 ± 0.5	12.6 ± 1.3	110.0 ± 1.1	3.6 ± 0.9	1.0

#### 3.2.2. NAPME–PVP-VA Formulations

**Figure 6:**
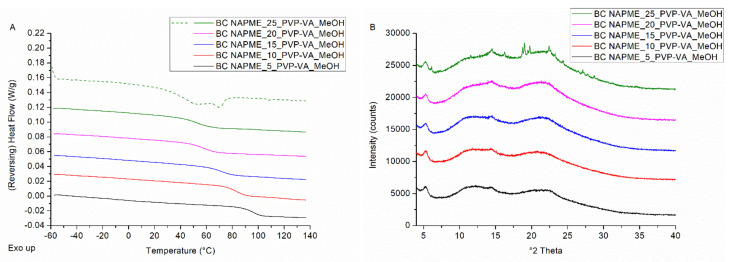
(**A**) mDSC thermograms of milled NAPME–PVP-VA-coated beads (BC), with drug loadings from 5% (black) to 25% (green) by intermediate steps of 5%. The THF (dashed) and RHF (solid) signals are shown as arbitrary units. (**B**) Corresponding XRPD diffractograms. The intensities are shown as arbitrary units.

In the next step, the ability of bead coating and spray drying to manufacture high drug-loaded ASDs was evaluated for the non-interacting drug–polymer model system NAPME–PVP-VA. The non-interacting behavior is confirmed with FTIR (see Figure S2 Supporting Information). The drug loading screening for NAPME–PVP-VA coated onto pellets resulted in the highest possible drug load of 20 wt%. For the formulation with 25% drug load, mDSC thermograms (Figure 6A) and XRPD diffractograms (Figure 6B) are characterized by a melting event and distinct Bragg peaks, respectively. XRPD and SEM analysis of the coated pellets as such also revealed the presence of NAPME crystals for the formulation with 25% drug load (data not shown).

**Figure 7:**
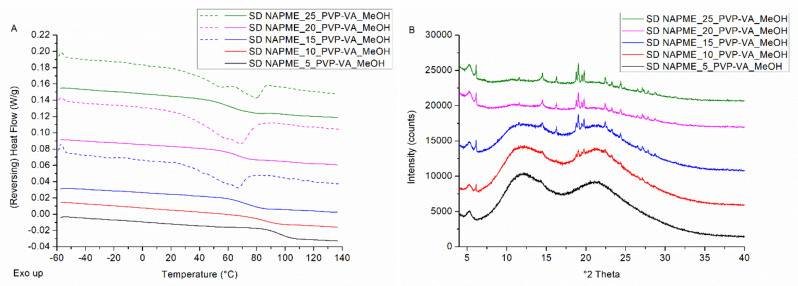
(**A**) mDSC thermograms of spray dried NAPME–PVP-VA systems (SD) with drug loadings from 5% (black) to 25% (green) by intermediate steps of 5%. The THF (dashed) and RHF (solid) signals are shown as arbitrary units. (**B**) Corresponding XRPD diffractograms, where the intensities are also shown as arbitrary units.

For the spray dried NAPME–PVP-VA systems, interestingly, the drug loading screening resulted in a maximum achievable drug weight fraction of only 5%. XRPD diffractograms presented in Figure 7B affirm the presence of NAPME characteristic Bragg peaks from 10% drug loading on. The mDSC thermograms (Figure 7A), however, point out the presence of crystalline content as only 15% of drug loading. This discrepancy can be explained by the coincidence of evaporation of considerable residual solvent amounts (see Table 3) with the NAPME melting events, of which the latter decrease with increasing PVP-VA fractions. This finding demonstrates the essentiality of applying complementary solid-state analysis techniques to correctly interpret the phase behavior of the ASD formulations [37].

Again, bead coating shows a greater ability to manufacture high drug-loaded ASDs, with a remarkable difference as compared to spray drying for the non-interacting drug–polymer system studied. The superiority of bead coating to incorporate higher drug weight fractions was also demonstrated in one of our previous studies, where non-interacting MIC–PVP-VA formulations were examined [15]. Achieving higher drug loads when coating non-interacting ASD systems is thus demonstrated for a low crystallization tendency compound (i.e., MIC), as well as for a compound exhibiting a rapid crystallization tendency (i.e., NAPME). The theoretical expectation that bead coating offers less kinetic stabilization than spray drying was thus again not met in practice, hence the abovementioned discussion (see Section 3.2.1) is also relevant in this case. For the NAPME–PVP-VA formulations, as well, slightly smaller differences between product temperature and T_g_ were obtained for spray drying because of the higher value of the T_outlet_ to the product T_g_, which might at least partially contribute to an increased rate of crystallization [34]. Distinct maximum achievable drug loads depending on the manufacturing technique applied have been frequently reported in the field of ASDs, although in most cases this involves a comparison between spray drying and hot melt extrusion [9,24,36,38]. Bhugra et al. evaluated spray drying and freeze drying and concluded that the selected preparation technique impacts the molecular mobility of the amorphous system [39].

It is also important to note that, in general, higher drug loadings can be obtained for the NAP–PVP-VA system as compared to the NAPME–PVP-VA system, which underpins the importance of drug–polymer interactions in achieving high drug-loaded ASDs. Indeed, these interactions are an additional stabilizing factor in ASDs, proved to result in higher miscibility and physical stability [4,8,40]. 

A detailed overview of the phase behavior of coated and spray dried NAPME–PVP-VA formulations is presented in Table 3. Again, T_g_ values decrease with increasing drug fractions. Contrary to what was found for NAP, T_g_ values of spray dried NAPME–PVP-VA systems are systematically higher than those obtained for the milled ASD-coated pellets, and this cannot be explained by the corresponding residual solvent levels. This might, again, at least partially, be attributed to small differences concerning NAPME content. Residual solvent percentages are markedly higher as compared to the NAP formulations, which can be ascribed to higher PVP-VA fractions in the NAPME formulations. T_g_ widths are quite comparable for the NAPME–PVP-VA ASDs coated onto pellets and their spray dried counterparts, and the T_g_ width also generally increases as the NAPME fraction increases, indicating more heterogeneity in the system.

### 3.3. Repeatability

Given the small difference in maximum achievable drug loads for the spray dried and coated NAP–PVP-VA systems and also with a view to the physical stability study, the repeatability of this outcome was assessed. More specifically, four batches of the same formulation were prepared with both manufacturing techniques, and the analysis results are summarized in Table 4. When NAP–PVP-VA with a drug weight fraction of 40% is deposited onto pellets, no melting endotherms could be detected with mDSC for any batch, and corresponding XRPD diffractograms did not show any NAP characteristic Bragg peaks. This corroborates the aforementioned definition of 40 wt% as the highest possible drug weight fraction. The small differences in T_g_ values observed between different batches are attributable to small differences concerning residual solvent levels. When NAP–PVP-VA with a drug weight fraction of 40% is spray dried, the results are less consistent. Only in one batch, XRPD revealed a NAP characteristic Bragg peak, and for three out of four batches, both mDSC and XRPD indicated the formation of an amorphous system. This finding implies that our aforementioned determination of 35 wt% as the maximum achievable drug load for spray dried NAP–PVP-VA systems needs to be nuanced. The inconsistency between distinct batches can be explained by the fact that the drug–polymer miscibility limit is approached at 40 wt% NAP. To illustrate, a drug weight fraction of 40% was reported as the highest possible for spray dried NAP–PVP-VA systems by Dedroog et al. [36]. Spray dried NAP–PVP-VA batches with a drug loading of 35 wt%, as part of the repeatability assessment, were found to be amorphous in all cases (data not shown). Therefore, spray dried formulations with 35%, as well as with 40% drug weight fractions (i.e., the batches that were found to be amorphous at time point zero), were included for physical stability assessment, of which the latter enables a direct comparison with the coated pellet formulation. As reported in Table 4, it can again be concluded that T_g_s are generally broader for the spray dried samples as compared to the ASDs deposited onto pellets, suggesting a greater extent of heterogeneity.

### 3.4. Physical Stability Study

**Figure 8:**
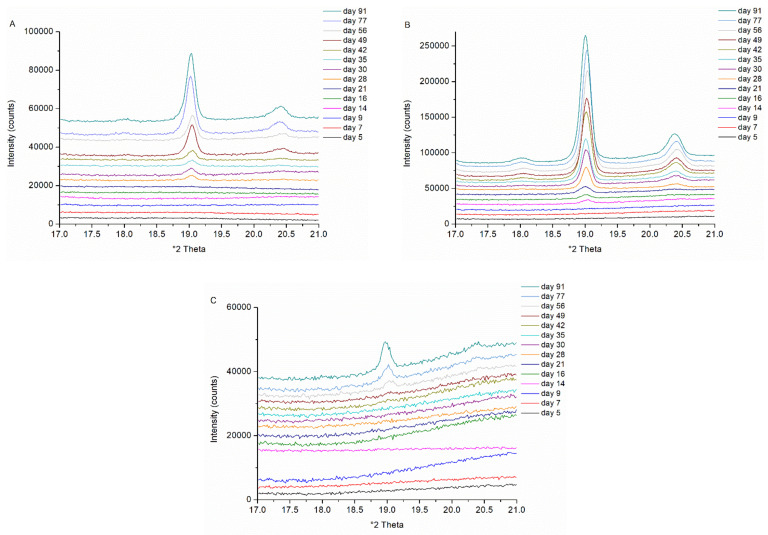
XRPD diffractograms for the various analysis time points (days upon storage) for (**A**) the NAP_40_PVP-VA formulation coated onto pellets (BC 40 milled), (**B**) the spray dried NAP_40_PVP-VA formulation (SD 40) and (**C**) the spray dried NAP_35_PVP-VA system (SD 35), stored at 40 °C/75% RH. Intensities are shown as arbitrary units.

To finalize the direct comparison between bead coating and spray drying, physical stability was assessed for the formulations with the highest possible NAP load per manufacturing technique (i.e., 40 wt% for bead coating and both 35 wt% and 40 wt% for spray drying). The days upon storage on which crystallinity could first be detected are summarized in Table 5 for the different storage conditions. These outcomes are based on XRPD analyses, since XRPD was found to be more sensitive than mDSC within the scope of the physical stability assessment. Recent studies also reported the greater sensitivity of XRPD as compared to mDSC and attributed this to dissolution of the drug in the polymer during the heating procedure of mDSC [13,41]. In general, crystallinity could first be detected for the harshest stability condition (i.e., 40 °C/75% RH), followed by the medium 40 °C/0% RH condition. For the spray dried samples however, the first indication of crystallinity virtually coincides for the two conditions at 40 °C, hence RH seemed to be not decisive considering nucleation. RH does, however, affect subsequent crystal growth, given the slower evolution of crystallinity percentages over time for the 40 °C/0% RH condition as compared to the 40 °C/75% RH condition (see Tables S1 and S2, Supporting Information, respectively). Samples stored at 4 °C/0% RH were still amorphous after three months of storage. It is also important to note the time lag between the first indication of crystallinity for milled coated pellets and coated pellets as such. As indicated in the drug loading screening part, XRPD could detect NAP crystals more readily in the milled samples than on coated beads as such, and in this regard SEM appeared to be a valuable solid-state characterization technique (see Section 3.2.1). Therefore, SEM was also implemented in this case to evaluate whether NAP crystals could be visualized on coated pellets as such around the time point at which XRPD first indicated NAP crystals in the milled sample. As a result, NAP crystals could be detected on coated pellets as such as of day 28 and as of day 42 on, for the 40 °C/75% RH and 40 °C/0% RH storage condition, respectively (data not shown). Hence, the XRPD results for milled coated pellets provide a good representation for the SEM outcomes of coated pellets as such. Despite the fact that XRPD was able to detect crystallinity on day 42 for the pellet formulation stored at 40 °C/0% RH, no NAP characteristic Bragg peaks could be observed for the timepoints thereafter (see Table S2, Supporting Information). This finding suggests a crystalline content close to the XRPD limit of detection, especially given the slow crystal growth process at 40 °C/0% RH. Interestingly, the NAP–PVP-VA systems with 40% drug loads coated onto pellets were found to be more physically stable than their 40% drug load spray dried counterparts. This finding most probably originates from the distinct extent of initial heterogeneity. As delineated in Table 4, T_g_ widths were generally larger for the spray dried samples as compared to those of coated ASDs. This outcome is, however, in contrast with the results of Paudel et al., who found that faster evaporation conditions generated spray dried solid dispersions with a wider miscibility gradient but with greater physical stability [31]. Furthermore, the superior physical stability of coated NAP–PVP-VA systems is more pronounced for the medium 40 °C/0% RH condition as compared to the harsh 40 °C/75% RH condition, i.e., a difference of approximately one month and two weeks, respectively. The spray dried formulations with 35% NAP weight fractions were physically the most stable, which is attributable to the presence of a larger polymer fraction, thus more stabilizer. To illustrate, XRPD diffractograms for the various analysis time points are shown in Figure 8, for all formulations investigated, stored at 40 °C/75% RH.

## 4. Conclusions

In this study, a direct comparison was made between bead coating and spray drying related to their ability to manufacture high drug-loaded ASDs in combination with a physical stability evaluation of the resulting formulations. For this purpose, two fast crystallizing model compounds were selected, namely, NAP and its methyl ester, resulting in an interacting and non-interacting drug–polymer model system in combination with PVP-VA, respectively. In the first step, a drug loading screening was performed for both model systems and both manufacturing techniques and the highest possible drug load that still results in an amorphous system was defined. For the interacting NAP–PVP-VA system, initially, a rather small difference in highest possible drug loading was observed for NAP–PVP-VA-coated ASDs in comparison to spray dried NAP–PVP-VA (i.e., 40 wt% vs. 35 wt%). Later on, the determination of 35 wt% as the maximum achievable drug load for spray dried NAP–PVP-VA systems was nuanced, abolishing the previously defined difference between bead coating and spray drying. Nevertheless, T_g_ widths were generally larger for the spray dried samples, implying that these systems are more heterogeneous in comparison to the ASDs deposited onto pellets. For the non-interacting NAPME–PVP-VA system, interestingly, bead coating showed greater ability to manufacture high drug-loaded ASDs as compared to spray drying (i.e., 20 wt% vs. 5 wt%). Moreover, the importance of drug–polymer interactions in achieving high drug loadings was demonstrated. In the next step, the interacting NAP–PVP-VA systems were further examined with respect to their physical stability for both manufacturing techniques. NAP_40_PVP-VA systems coated onto pellets were found to be more physically stable than their spray dried counterparts, which can most probably be explained by the distinct extent of initial heterogeneity. This study contributed to an in-depth understanding of the underexplored bead coating process and ultimately intensified its value in relation to spray drying for ASD manufacturing purposes.

## Figures and Tables

**Table 1:** Average crystallinity percentages (%) for NAP and NAPME spray dried from MeOH ± standard deviation (sd).

	NAP	NAPME
Day 0	97.31 ± 1.18	94.30 ± 3.18
Day 1	96.21 ± 3.50	97.93 ± 0.68
Day 7	96.16 ± 1.96	96.82 ± 0.94

**Table 3:** Overview of the phase behavior of NAPME–PVP-VA formulations prepared with bead coating and spray drying. Average T_g_s, T_g_ widths, T_m_ values and crystallinity percentages ± sd are obtained from mDSC measurements. Residual solvent percentages are derived from TGA analyses. NA = not applicable.

Drug Load	Average T_g_ ± sd (°C)	Average T_g_ Width ± sd (°C)	Average T_m_ ± sd (°C)	Average Crystalline Content ± sd (%)	Residual Solvent (%)
Bead coating					
5%	95.3 ± 0.03	13.4 ± 0.2	NA	NA	4.4
10%	81.8 ± 1.6	13.2 ± 1.4	NA	NA	3.3
15%	69.1 ± 2.1	19.2 ± 3.6	NA	NA	3.4
20%	57.4 ± 1.3	17.9 ± 0.8	NA	NA	3.1
25%	53.2 ± 1.7	21.0 ± 2.3	64.9 ± 0.6	4.5 ± 0.4	2.9
Spray drying					
5%	95.8 ± 0.8	13.9 ± 2.4	NA	NA	4.5
10%	85.3 ± 0.3	17.9 ± 0.1	NA	NA	3.7
15%	73.1 ± 1.3	18.5 ± 3.6	62.5 ± 0.6	9.5 ± 1.4	3.4
20%	64.2 ± 4.1	17.5 ± 5.6	63.1 ± 1.2	12.6 ± 3.5	2.8
25%	55.8 ± 5.6	20.4 ± 1.2	66.7 ± 2.0	16.4 ± 2.8	3.2

**Table 4:** Phase behavior of NAP–PVP-VA formulations with 40% drug load prepared with bead coating and spray drying, as part of the repeatability assessment. Average T_g_s, T_g_ widths, T_m_ values and crystallinity percentages ± sd are obtained from mDSC measurements. Residual solvent percentages are derived from TGA analyses. NA = not applicable. Data marked in light grey are the results from the initial drug loading screening (see, also, Table 2) and are shown as comparison.

Average T_g_ ± sd (°C)	Average T_g_ Width ± sd (°C)	Average T_m_ ± sd (°C)	Average Crystalline Content ± sd (%)	Residual Solvent (%)	NAP Characteristic Bragg Peaks on Diffractogram?
Bead coating					
58.4 ± 0.3	11.4 ± 0.6	NA	NA	1.0	No
54.5 ± 1.8	13.4 ± 0.5	NA	NA	1.7	No
56.6 ± 0.5	11.0 ± 1.9	NA	NA	1.5	No
58.0 ± 0.4	11.4 ± 0.3	NA	NA	1.2	No
58.5 ± 0.3	9.8 ± 1.2	NA	NA	1.2	No
Spray drying					
55.2 ± 2.0	17.1 ± 0.4	93.5 ± 1.4	1.1 ± 0.2	1.3	Yes
55.6 ± 0.8	16.3 ± 0.4	NA	NA	1.4	Yes
54.8 ± 0.9	16.0 ± 1.2	NA	NA	1.3	No
54.7 ± 0.9	15.4 ± 1.2	NA	NA	1.0	No
56.6 ± 0.7	17.6 ± 1.9	NA	NA	1.5	No

**Table 5:** Overview of the days upon storage on which crystallinity could first be detected with XRPD, for the NAP_40_PVP-VA formulation coated onto pellets (BC 40), the spray dried NAP_40_PVP-VA formulation (SD 40) and the spray dried NAP_35_PVP-VA system (SD 35), stored at 4 °C/0% RH, at 40 °C/0% RH and at 40 °C/75% RH. Grey boxes imply that no NAP characteristic Bragg peak could be detected after three months of storage.

Sample	First Indication of Crystallinity (Based on XRPD)		
	4 °C/0% RH	40 °C/0% RH	40 °C/75% RH
BC 40 (milled)		Day 42	Day 28
BC 40 (beads as such)			Day 42
SD 40		Day 9	Day 14
SD 35		Day 49	Day 49

## Supplementary Materials

The following supporting information can be downloaded at: https://www.mdpi.com/article/10.3390/pharmaceutics14030613/s1, Detailed synthesis procedure of NAPME, as well as corresponding NMR and HRMS data and a DSC purity analysis, Figure S1: FTIR spectra of crystalline NAP, PVP-VA, a physical mixture (PM) containing 35 wt% NAP and 65 wt% PVP-VA and a spray dried formulation (SD) consisting of 35 wt% NAP and 65 wt% PVP-VA, Figure S2: FTIR spectra of crystalline NAPME, PVP-VA, a physical mixture (PM) containing 5 wt% NAPME and 95 wt% PVP-VA and a spray dried formulation (SD) consisting of 5 wt% NAPME and 95 wt% PVP-VA, Table S1: Crystallinity percentages (i.e., degree of crystallinity relative to pure NAP) calculated for the various analysis time points (days upon storage) for the NAP_40_PVP-VA formulation coated onto pellets (BC 40 milled), the spray dried NAP_40_PVP-VA formulation (SD 40) and the spray dried NAP_35_PVP-VA system (SD 35), stored at 40 °C/75% RH, Table S2: Crystallinity percentages (i.e., degree of crystallinity relative to pure NAP) calculated for the various analysis time points (days upon storage) for the NAP_40_PVP-VA formulation coated onto pellets (BC 40 milled), the spray dried NAP_40_PVP-VA formulation (SD 40) and the spray dried NAP_35_PVP-VA system (SD 35), stored at 40 °C/0% RH.
