# Supplementary material for: The Value of Bead Coating in the Manufacturing of Amorphous Solid Dispersions: A Comparative Evaluation with Spray Drying
Source: Pharmaceutics. 2022 Mar 11;14(3):613. doi: 10.3390/pharmaceutics14030613 (PMC8955898; doi:10.3390/pharmaceutics14030613)
Supplement: Supplementary file 1 [file pharmaceutics-14-00613-s001.zip › pharmaceutics-1610222-supplementary.pdf]

pharmaceutics

MDPI

Supplementary Materials: The Value of Bead Coating in the Manufacturing of Amorphous Solid Dispersions: A Comparative Evaluation with Spray Drying

Eline Boel, Felien Reniers, Wim Dehaen and Guy Van den Mooter

1. *Synthesis of methyl 2-(6-Methoxynaphthalen-2-yl)propanoate (i.e., naproxen methyl ester)*

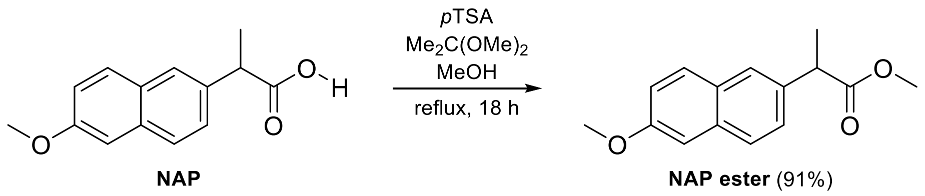

2-(6-Methoxynaphthalen-2-yl)propanoic acid (50 g, 217 mmol), *p*-toluenesulfonic acid (323 mg, 1.70 mmol), 2,2-dimethoxypropane (32 mL, 261 mmol), and MeOH (217 mL) were added to a round bottom flask. The reaction mixture was refluxed for 18 h, followed by cooling to -20 °C. The formed crystals were filtered off and three times washed with cold MeOH. The pure product was obtained as a white solid in 91 % yield (48 g).

NMR spectra were acquired on commercial instruments (Bruker AMX 400 MHz) and chemical shifts (δ) are reported in parts per million (ppm) referenced to the internal (NMR) solvent signal. High-resolution mass spectra were acquired on a quadrupole orthogonal acceleration time-of-flight mass spectrometer equipped with an APCI source (Synapt G2 HDMS, Waters, Milford, MA, USA). Samples were infused at 3 μL/min and spectra were obtained in positive mode with a resolution of 15 000 (FWHM) using leucine enkephalin as lock mass.

^{1}H NMR (400 MHz, $CDCl_3$) *δ* (ppm) 7.73 (s, 1H), 7.70 (s, 1H), 7.69 – 7.66 (m, 1H), 7.45 – 7.38 (m, 1H), 7.19 – 7.10 (m, 2H), 3.91 (s, 3H), 3.87 (q, *J* = 7.2 Hz, 1H), 3.68 (s, 3H), 1.59 (d, *J* = 7.2 Hz, 3H).
^{13}C NMR (101 MHz, $CDCl_3$) *δ* (ppm) 175.2, 157.7, 135.8, 133.8, 129.4, 129.0, 127.3, 126.3, 126.0, 119.1, 105.7, 55.4, 52.1, 45.5, 18.7.
HRMS (ESI^+) m/z calculated for $[M+Na]^+$: 267.0992, found: 267.0992.

NMR spectra:

^{1}H NMR (400 MHz, $CDCl_3$)

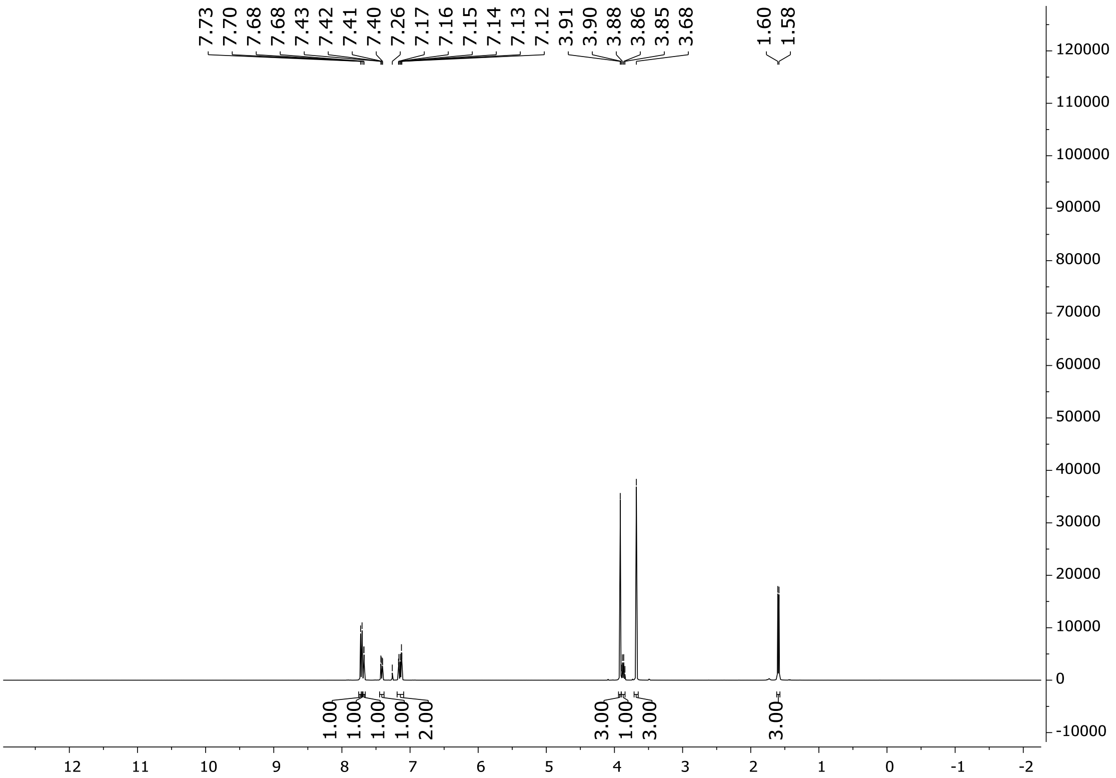

^{13}C NMR (101 MHz, $CDCl_3$)

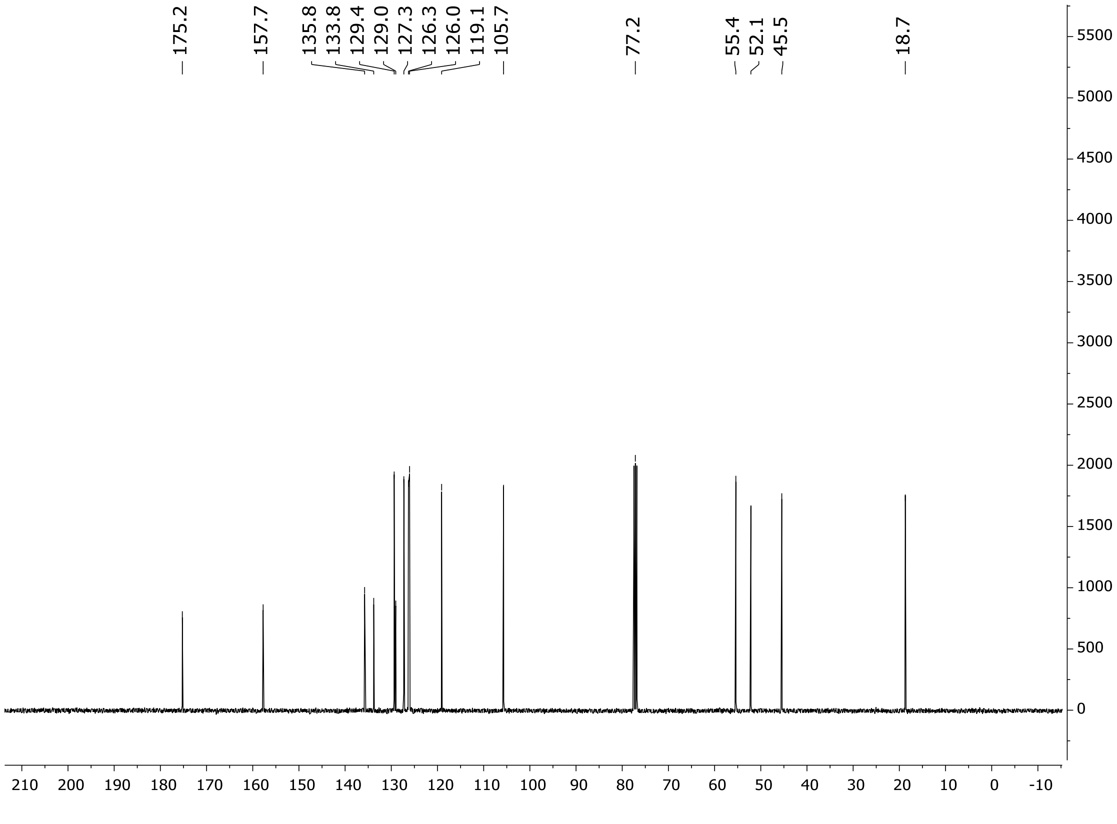

DSC purity analysis (ASTM E928) NAPME:

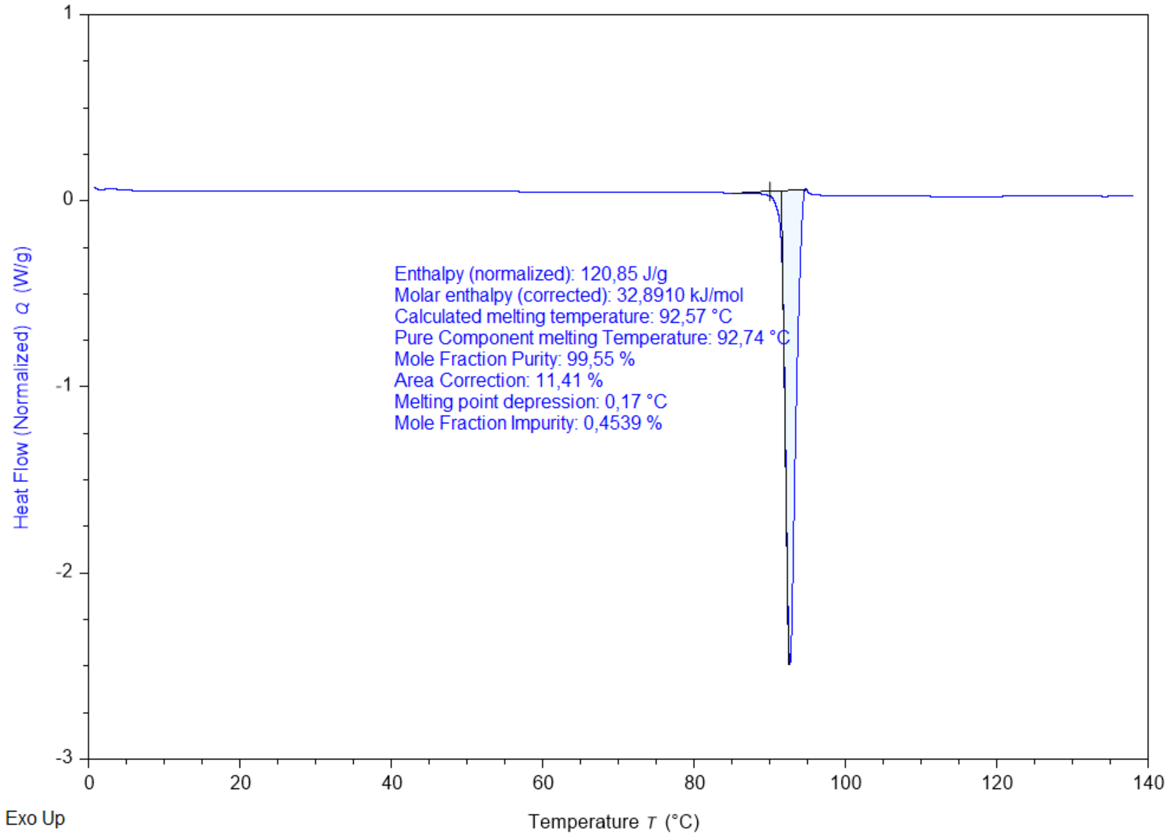

2. *FTIR spectra*

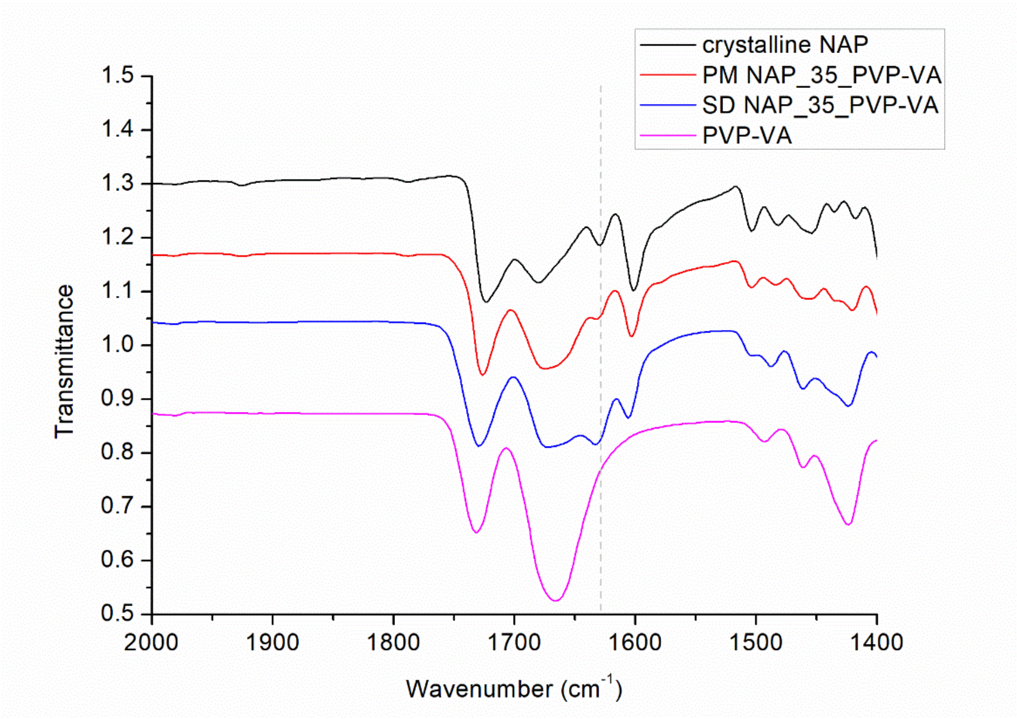

Figure S1. FTIR spectra of crystalline NAP, PVP-VA, a physical mixture (PM) containing 35 wt% NAP and 65 wt% PVP-VA and a spray dried formulation (SD) consisting out of 35 wt% NAP and 65 wt% PVP-VA.

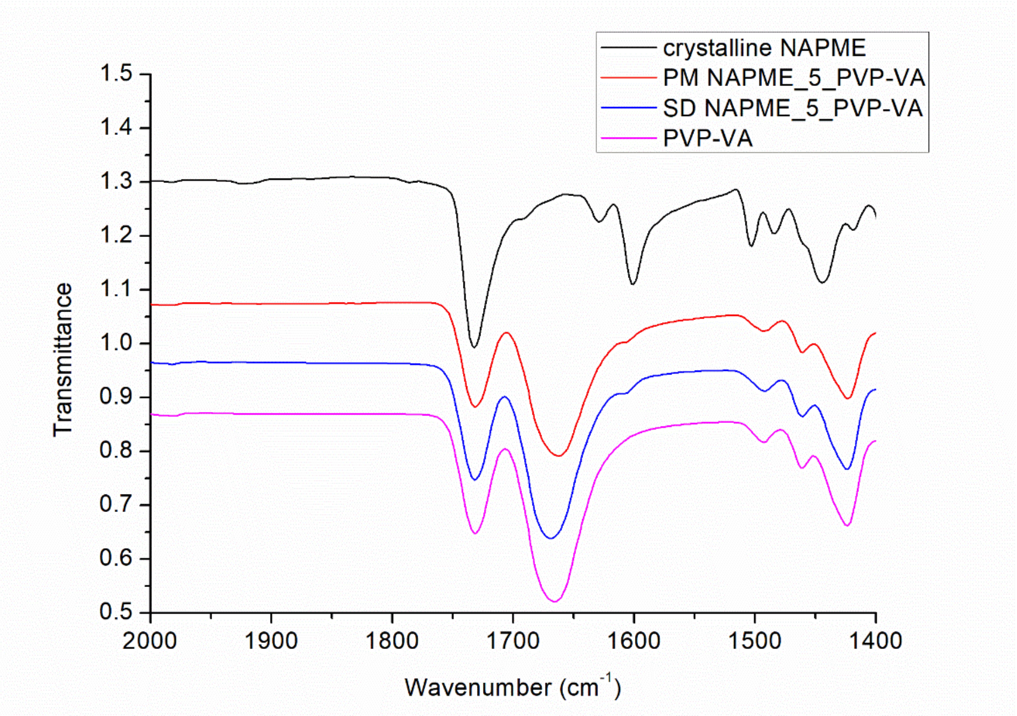

Figure S2. FTIR spectra of crystalline NAPME, PVP-VA, a physical mixture (PM) containing 5 wt% NAPME and 95 wt% PVP-VA and a spray dried formulation (SD) consisting out of 5 wt% NAPME and 95 wt% PVP-VA.

3. *Physical stability study*

Table S1. Crystallinity percentages (i.e., degree of crystallinity relative to pure NAP) calculated for the various analysis time points (days upon storage) for the NAP_40_PVP-VA formulation coated onto pellets (BC 40 milled), the spray dried NAP_40_PVP-VA formulation (SD 40) and the spray dried NAP_35_PVP-VA system (SD 35), stored at 40 °C/75 % RH. Crystallinity percentage calculations were performed based on the AUC of the highest intensity Bragg peak, normalized by weight. Hatched boxes imply that no NAP characteristic Bragg peak could be detected.

Days upon storage at 40 °C/75 % RH	Relative crystallinity percentages (%)		
	BC 40 milled	SD 40	SD 35
Day 5			
Day 7			
Day 9			
Day 14		0.24	
Day 16		0.39	
Day 21		0.72	
Day 28	0.85	5.89	
Day 30	0.49	3.13	
Day 35	1.22	4.59	
Day 42	2.32	3.72	
Day 49	5.41	6.95	0.03
Day 56	2.51	6.88	0.07
Day 77	6.98	6.47	0.18
Day 91	6.70	5.81	0.27

Table S2. Crystallinity percentages (i.e., degree of crystallinity relative to pure NAP) calculated for the various analysis time points (days upon storage) for the NAP_40_PVP-VA formulation coated onto pellets (BC 40 milled), the spray dried NAP_40_PVP-VA formulation (SD 40) and the spray dried NAP_35_PVP-VA system (SD 35), stored at 40 °C/0 % RH. Crystallinity percentage calculations were performed based on the AUC of the highest intensity Bragg peak, normalized by weight. Hatched boxes imply that no NAP characteristic Bragg peak could be detected.

Days upon storage at 40 °C/0 % RH	Relative crystallinity percentages (%)		
	BC 40 milled	SD 40	SD 35
Day 5			
Day 7			
Day 9		0.14	
Day 14		0.13	
Day 21		0.50	
Day 28		0.40	
Day 35		0.94	
Day 42	0.14	0.65	
Day 49		0.70	0.05
Day 56		1.25	0.07
Day 77		1.07	0.12
Day 84		0.91	0.15
Day 91	0.06	0.75	0.14
